# Novel hydration and nutritional strategies for sickle cell disease

**DOI:** 10.1002/jha2.9

**Published:** 2020-05-06

**Authors:** Marcy C. Purnell, Michong Rayborn

**Affiliations:** ^1^ School of Leadership and Advanced Nursing Practice The University of Southern Mississippi Hattiesburg Mississippi

**Keywords:** electrolytes, glucose, perioperative management, sickle cell disease, sickle cell trait, vaso‐occlusive crises

## Abstract

**Introduction:**

Sickle cell disease and sickle cell trait affect over 300 million people worldwide. Vaso‐occlusive crises (VOCs) are the most common reason that these patients seek medical care.

**Objectives:**

Recently, a newly identified “trigger” (involving glucose and electrolytes) for a mechanism of abnormal actin polymerization may offer further understanding with regard to the sequence of events that cascade to complications such as VOCs in those with sickle cell disease (SCD) and as well as those with sickle cell trait.

**Methods:**

A literature review to identify the current standard of care guidelines for hydration and nutritional strategies during VOCs in patients with SCD and sickle cell trait was conducted in PubMed, OVID, and Google Scholar.

**Results:**

This review suggested that current rationales for hydration and nutritional strategies for these patients during periods of crisis are generally based on consensus and have remained largely undefined to date.

**Conclusion:**

This new trigger, along with this literature review, suggests investigations related to serum glucose and cation (electrolyte) levels may help define novel strategies for the development of protocols/standard of care with regard to intravenous and oral hydration/nutritional guidelines in these patients during both clinical and perioperative management periods.

## 1 INTRODUCTION

Sickle cell disease (SCD) is an autosomal recessive disorder and affects approximately 30 million people worldwide with the highest occurrences being among individuals of African, Middle Eastern, and Central Indian ancestry.^1^ In many endemic areas where resources are insufficient, most individuals with SCD die before reaching adulthood.^2^ Also, sickle cell trait, which is even more common than SCD, occurs in approximately 300 million people worldwide and can also be associated with significant morbidity and mortality.^3^ In 2016, inpatient stays for SCD alone in the United States totaled $811.4 million with a 5‐day average length of stay.^4^ Sickle cell disease patients undergo both emergent and elective surgical procedures involving SCD complications as well as surgeries for indications that are common to the general population. The data suggest that these patients experience higher perioperative complication rates than other populations related to vaso‐occlusive crises (VOCs) such as acute chest syndrome (ACS), postoperative infection, congestive heart failure, and cerebrovascular accidents.^5^ Fluid/hydration therapies used in the management of these SCD and sickle cell trait patients both in the clinical and perioperative arenas have been poorly defined possibly due to a lack of understanding of the cascade of events that lead to the “trigger” for VOCs. Presently, the initial management decisions for hydration and nutritional management of these patients are made on consensus.^6^ Recently, a newly identified “trigger” of increased levels of glucose and cation (electrolyte) levels and the subsequent abnormal actin polymerization (in the spectrin‐actin complex in cytoskeleton of the red blood cell membrane) may offer further understanding with regard to the sequence of events that can lead to crisis in those with SCD and sickle cell trait and may help define novel strategies for the development of treatment protocols/standard of care with regard to IV and oral hydration/nutritional guidelines in these patients during both clinical and perioperative management.^7^ This review is directed toward the global audience of general physicians, anesthetists, advanced practice practitioners, nurses and intensivists involved in the collaborative clinical and perioperative management of SCD, and sickle cell trait patients.

## 2 NEW TRIGGER MECHANISM IN SICKLE CELL DISEASE

In 1949, there was a discovery of the abnormal sickle protein (HbS) mutation in SCD where the β‐globin chains were found to have a valine in the place of glutamic acid (β6Glu→Val).^8^ In SCD patients, valine replaces glutamic acid on *both* β‐globin chain subunits on the hemoglobin protein, while in sickle cell trait patients this substitution occurs on only *one* of the β‐globin chain subunits. This was an important discovery, but it has not led to an explanation for the cascade of events that occur in VOCs and other complications in SCD and sickle cell trait patients. The recently identified and published “triggers” of glucose and cations/electrolytes that may spark the cascade of events that lead to an abnormal polymerization of the actin protein in the cytoskeleton, sickling of the red blood cells and vasocclusive crises, may also offer some new insights into hydration/nutritional strategies as well as further research studies for both the clinical and perioperative management in these patients^7^:

(1) $$\left(↑\mathrm{Glucose}\to ↑\mathrm{HbS}\left(\mathrm{Val}\right)\mathrm{glycation}\right)+\left(↑\mathrm{Electrolytes}\right)=\mathrm{VOCs}$$

This new finding suggests that the substitution of β6Glu→Val on the HbS molecule (each red blood cell contains ∼270 million hemoglobin molecules) along with the increased and irreversible glycation (associated with increased levels of serum glucose) of these extra mutant hydrophobic valines in SCD and in sickle cell trait may ultimately increase the subsequently generated ATP‐ATPase availability and activity via Amadori‐mediated pyruvate kinase activity.^7^ When elevated levels of serum cations/electrolytes (Mg^+^, Ca^+^, Na^+^, K^+^) are added to this increased ATP availability, this combination may increase the ATP G‐actin concentration that leads to a critical concentration threshold that allows for polymerizations of the both the positive and negative ends of the F‐actin filaments.^7^ Surpassing this critical concentration may lead to an abnormal elongation (polymerization) of stiff actin filaments (actin filaments must maintain a uniform length of ∼37 ηm to ensure cytoskeletal membrane integrity/function) that ultimately disrupts the spectrin‐actin cytoskeletal network, band 3/AE1 anion channels, and aquaporin channels, thereby beginning a cascade of events that lead to dehydration/loss of neutrality/loss of function/catastrophic failure of the red blood cell = Sickled RBCs.^7^

Sickle cell patients are known to develop VOCs under periods of stress (which often lead to hyperglycemic states) such as anxiety, emotional stress, dehydration, acidosis, hypoxia, vascular stasis, and increased blood viscosity, and during perioperative period.^9^, ^10^ These crises often lead to pain, ACS, infections, organ failure, and death.

In sickle cell trait, as stated earlier, the individual has one abnormal allele of the hemoglobin β gene and therefore does not have the whole burden of the disease (which may be possibly due to fewer available valines and therefore a smaller increase in glycation and ATP‐ATPase availability, etc.). Even without the whole burden of SCD, the evidence shows that sickle cell trait is associated with morbidities such as hematuria, renal papillary necrosis, hyposthenuria, splenic infarction, exertional rhabdomyolosis, and exercise‐related sudden death.^3^ It appears that there may also be an independent association of sickle cell trait with hyphemia, venous thromboembolic events, fetal loss, neonatal deaths, preeclampsia, and possibly ACS and anemia in pregnancy.^3^

Since fluid therapy guidelines (ie, what type of fluid, rate, maintenance vs bolus, etc.) in SCD and possibly in those patients with sickle cell trait have been poorly defined to date, we may begin to look at how this newly defined polymerization “trigger” may help to advance the knowledge needed to begin to reveal possible novel treatment protocols/standards of care and possible improvement in clinical outcomes for these patients.^6^, ^11^

## 3 CURRENT HYDRATION AND NUTRITIONAL STRATEGIES IN SICKLE CELL DISEASE

Intravenous fluid (IVF) and oral hydration/nutrition are an important part of the current standard of care therapy during both clinical and perioperative periods in patients experiencing VOCs with SCD and sickle cell trait.^12^, ^13^ However, the specific type of IVF/hydration/diet to administer during VOCs continues to remain controversial and unclear. Regarding IVF administration, some clinicians use normal saline (NS), an isotonic IVF, during treatment for VOCs, others disagree with the use of NS due to possible issues related to potential inability to secrete sodium with the hyperosmolar load that patients with SCD‐related renal dysfunction may develop.^11^, ^14^ Therefore, other clinicians recommend administering hypotonic IVFs during VOCs.^15^, ^16^ Rosa et al reported in a limited study with three SCD patients that maintaining hyponatremia (ie, Na = 120–125 mEq/L) reduced the frequency of VOCs compared with patients treated in the current standard of care model.^17^ Another study of seven patients reported improvement in pain and number of circulating sickle cells after rapid administration of the hypotonic IVF ½ NS (ie, Na = 77 mEq/L, Table 1). Consequently, the investigators also expressed concerns for this strategy due to the lack of ability to sustain these low sodium levels due to risk of seizures and therefore has prohibited the widespread use of this protocol to date.^18^ Recently, Dr Marcus Carden and colleagues conducted a multiinstitution retrospective cohort study of the use of supplemental fluids for acute VOCs in 20 pediatric emergency departments evaluating outcomes of 400 children (median age, 13.8 ± 4.9 years) with SCD. The majority (66%) of patients were administered a hyperosmolar NS fluid bolus (in those with no signs of dehydration) with an average volume of 18.2 ± 9.5 mL/kg. These patients who received this hyperosmolar NS fluid bolus reported/experienced smaller improvements in pain scores, spent more time in the emergency department, and had higher rates of readmissions.^11^

**TABLE 1:** Common IV fluids

Name	pH	Ingredients in1 L	Normal osmolarity (240‐340 mOsm/L)	Type of solution
D5W 5% Dextrose In water	5.0	5 g dextrose (170 calories/L)	252 mOsm/L	Isotonic (hypotonic in body)
NS 0.9% Sodium chloride	5.7	154 mEq Sodium 154 mEq Chloride	308 mOsm/L	Isotonic
LR Lactated Ringer's solution	6.6	130 mEq Sodium 4 mEq Potassium 3 mEq Calcium 109 mEq Chloride 28 mEq Sodium lactate	273 mOsm/L	Isotonic
½ NS 0.45% Sodium chloride	5.6	77 mEq Sodium 77 mEq Chloride	154 mOsm/L	Hypotonic
3% Sodium chloride	5.0	513 mEq Sodium 513 mEq Sodium	1026 mOsm/L	Hypertonic
5% Sodium chloride	5.8	855 mEq Sodium 855 mEq Sodium	1710 mOsm/L	Hypertonic
D10W 10% Dextrose in water	4.3	10 g Dextrose (340 calories/L)	505 mOsm/L	Hypertonic
D5 ¼NS Dextrose ¼ (0.25%) Saline	4.4	5 g Dextrose 77 mEq Sodium 77mEq Chloride	406 mOm/L	Hypertonic
D5 ½ NS 5% Dextrose in 0.45 sodium chloride	4.4	5 grams Dextrose 77 mEq Sodium 77 mEq Chloride	406 mOsm/L	Hypertonic
D5NS 5% Dextrose in normal saline	4.4	5 g Dextrose 154 mEq Sodium 154 mEq Chloride	560 mOsm/L	Hypertonic
D5LR 5% Dextrose in normal saline	4.9	5 g Dextrose (170 calories/L) 130 mEq Sodium 4 mEq Potassium 3 mEq Calcium 109 mEq Chloride 28 mEq Sodium Lactate	525 mOsm/L	Hypertonic

With regard to oral hydration, current NHLBI guidelines suggest encouraging oral hydration, along with analgesics, during VOCs and to give IVF only when/if the patient is unable to drink or take liquids by mouth (as in the perioperative period or critical care periods). Also, the AANA's (2017) Enhanced Recovery after Surgery (ERAS) guidelines recommend a carbohydrate beverage (up to 2 h preoperatively) for all surgical patients in the preoperative phase (no differentiation/customization for populations such as sickle cell etc.).^19^ Despite the lack of clear evidenced‐based guidelines, clinicians continue to use large IV volumes of NS and crystalloids and concentrated glucose solutions given over a short period of time.^19^, ^20^ Carden and colleagues reported that 83.2% of clinicians frequently/always use isotonic crystalloid bolus (NS or lactated Ringer's solution), while 36.9% frequently/always use isotonic/crystalloid at a maintenance rate (Table 2).^20^ They also reported a low rate of 10.2% use of hypotonic fluid (½ NS) for maintenance (Table 2).^20^

**TABLE 2:** Use of IV fluids during adult VOE as administered by attending, advanced practice providers, residents, and medical students in ED^20^

Variable	Result	
Number of respondents	N = 244	
Type of provider	Attending	74.1%
	Advanced practice practitioner	3.3%
	Resident Medical student Other number	19.7% 1.0% 1.6%
Type of fluids and rate of administration given	Isotonic crystalloid bolus	
	Frequently/always	83.2%
	Never/rarely	16.4%
	Isotonic crystalloid maintenance	
	Frequently/always	36.9%
	Never/rarely	57.8%
	Hypotonic maintenance	
	Frequently/always	10.2%
	Never/rarely	88.9%

Sickle cell patients are also thought to be continually hypovolemic due to hyposthenuria or a renal condition that decreases their ability to concentrate their urine.^21^ But aggressive hydration of these patients to hypervolemic states (with bolus administrations) may be harmful due to the increased risk of the development of pulmonary edema, hypoxia, dyspnea, and atelectasis. Atelectasis is thought to be a major risk factor in the development of ACS, which is responsible for ∼25% of sickle cell deaths during hospitalizations.^22^

## 4 NOVEL HYDRATION AND NUTRITIONAL CONSIDERATIONS IN SICKLE CELL DISEASE

As reported above, earlier studies have highlighted fluid replacement strategies to maintain red blood cell integrity by inhibition of the efflux of intracellular K^+^, Cl^−^, and free water.^17^ Some have also focused on the blocking of Ca^2+^ activated K^+^ channel and the K^+^/Cl^−^ channels in the RBC membrane with limited success.^23^ Due to the recently identified potential polymerization trigger involving the influence of glucose and cation/electrolyte levels on the actin cytoskeletal protein in patients with SCD and sickle cell trait, it appears that attending to the foundational basics of fluid and electrolyte balance and serum glucose management may be critical components in the perioperative and clinical management of these patients. There are increased challenges with managing SCD and possibly sickle cell trait due to the high incidence of elevated sugar and salt intake that occurs in modern society today.^24^ Stressful events/illness are also known to both elevate serum blood glucose as well as challenge fluid and electrolyte balance. While the aforementioned studies were limited due to small sample sizes, they suggest that low normal or hyponatremic states may reduce pain and/or VOCs in some SCD patients.^17^, ^18^ This evidence suggests that cautious maintenance with hypotonic IV solution (½ NS) while keeping a close eye on serum glucose and sodium (as well as other electrolytes) levels (instead mostly relying on isotonic, isotonic crystalloids, and hypertonic solutions) may offer additional options when a clinician is ordering IVF hydration in both SCD and sickle cell trait.

These novel hydration and nutritional considerations appear to be in contrast to the current hydration/nutritional approaches that were recently reported by majority of clinicians and consist of using a bolus isotonic crystalloid solutions or hypertonic NS as a first‐line choice for VOCs (Table 2).^11^, ^20^ It should be restated that careful monitoring of sodium levels may be paramount in order to watch for severe hyponatremia with this approach. Also, the rate of IV fluid hydration needs to be carefully considered with regard to organ function/failure status in both the young and the aged as certain clinical presentations such as hypovolemic shock may dictate bolus isotonic crystalloid fluid administration, etc., while other patients may need other customized care choices.

Ultimately, careful studies need to be conducted to determine if and at what rate the infusion of hypotonic ½ NS may be indicated in order to reduce the polymerization trigger, hypervolemic states, and prolonged hyponatremic states as well as the development of associated complications of pulmonary edema and ACS. Also, studies on the tight control of blood glucose during the clinical and perioperative periods are also warranted.

Finally, but just as importantly, studies on controlling sugar and salt intake via oral hydration/nutritional intake may also be a critical component in the management of these patients. Fluid balance may need careful monitoring in both the clinical and perioperative management due to both the disease process and inability to eat/drink prior to operative/fasting procedures. Low glycemic index foods may be considered along with free water (limiting sugar, sugar substitutes, salt, and salt substitutes) in order to avoid increasing serum electrolytes and serum blood glucose (causing abnormal actin polymerization).

## 5 DISCUSSION

To date, the approach to treat VOCs has been more experience based rather than evidence based. Studies have suggested that hypotonic solutions (maintenance vs bolus) may offer improved outcomes for patients but there is concern for possible adverse effects with prolonged hyponatremic states.^17^, ^18^ Also, investigations have suggested that there may be poor outcomes with the use of isotonic and hypertonic NS and crystalloid boluses in VOCs (except in the cases of severe hypovolemic/shock/emergent states).^11^, ^20^ It appears that the newly identified molecular modeling trigger of actin polymerization (due to increased glycation in the presence of increased cations/electrolytes over the critical concentration for F‐actin elongation) may warrant future clinical investigations to develop consistent and efficacious hydration and nutritional guidelines along with ERAS guidelines during clinical and perioperative management for this challenging disease (Table 3).^7^, ^20^

**TABLE 3:** New considerations for clinical and perioperative management of SCD and sickle cell trait

**Guidelines**
Maintain euvolemia
Monitor sodium (Na+), Calcium (Ca+), Potassium (K+), and Magnesium (Mg+) levels closely.
Avoid hypernatremia, hypercalcemia, hyperkalemia, and hypermagnesemia.
Monitor serum blood glucose closely.
Maintain strict blood glucose control.
